# Laparoscopic Extirpation of a Schwannoma in the Lateral Pelvic Space

**DOI:** 10.1155/2016/1351282

**Published:** 2016-11-09

**Authors:** Eiji Hidaka, Yasuhiro Ishiyama, Chiyo Maeda, Kenta Nakahara, Shoji Shimada, Shumpei Mukai, Naruhiko Sawada, Fumio Ishida, Shin-ei Kudo

**Affiliations:** Digestive Disease Center, Showa University Northern Yokohama Hospital, 35-1 Chigasaki-chuou, Tsuzuki-ku, Yokohama 224-8503, Japan

## Abstract

Schwannomas in the lateral pelvic space are very rare. Here, we report the case of a 48-year-old woman who had a tumor detected in her abdomen by abdominal ultrasonography. Abdominal computed tomography and magnetic resonance imaging revealed a well-defined solid tumor of 65 mm in diameter in the right lateral pelvic space. We performed laparoscopic surgery under a diagnosis of a gastrointestinal tumor or neurogenic tumor. The tumor was safely dissected and freed from the surrounding tissues using sharp and blunt maneuvers. The tumor originated from the right sciatic nerve. Complete laparoscopic extirpation was performed with preservation of the right sciatic nerve. Pathological examination suggested schwannoma. The patient recovered well but had remaining sciatic nerve palsy in her right foot. Laparoscopic extirpation for a schwannoma in the lateral pelvic space was safe and feasible due to the magnified surgical field afforded by laparoscopy.

## 1. Introduction

Schwannomas are neurogenic tumors originating in the Schwann cells of the nerve sheath. These tumors generally occur in the head, neck, and extremities, and occurrence in the pelvic space is rare [1, 2]. There are a few reports of laparoscopic surgery (LS) for pelvic schwannomas [3, 4]. LS for schwannomas in the lateral pelvic space has not been reported. Recently, LS for the dissection of the lateral pelvic lymph nodes for locally advanced rectal cancers has been accepted in Japan. Several studies have reported that LS is safe and feasible for lateral lymph node dissection [5]. This report contains details of the successful use of laparoscopic extirpation of a schwannoma in the lateral pelvic space.

## 2. Case Presentation

**Figure 1:**
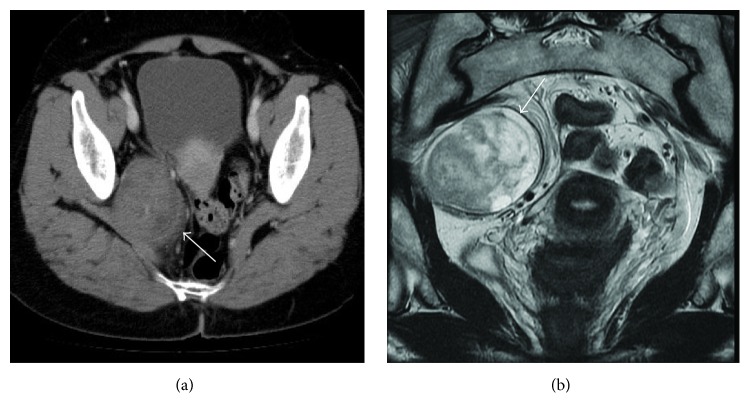
(a) Enhanced abdominal computed tomography revealing a 65 × 50 mm, solid, well-defined, heterogeneous mass (arrow) in the right lateral pelvis space. (b) Magnetic resonance imaging revealing heterogeneous hyperintensity in the tumor (arrow) on T2-weighted image.

A 48-year-old woman was admitted to our hospital with a mass in the pelvic space that was detected on abdominal ultrasonography (US). She had no past or family history of note. She had mild numbness in the right leg. Enhanced abdominal computed tomography (CT) revealed a 65 × 50 mm, solid, well-defined, heterogeneous mass in the right lateral pelvis space (Figure 1(a)). Magnetic resonance imaging of the tumor revealed heterogeneous hyperintensity on T2-weighted images (Figure 1(b)). The preoperative diagnosis was a gastrointestinal stromal tumor or a neurogenic tumor in the right lateral pelvic space. We performed laparoscopic extirpation of the tumor as follows.

**Figure 2:**
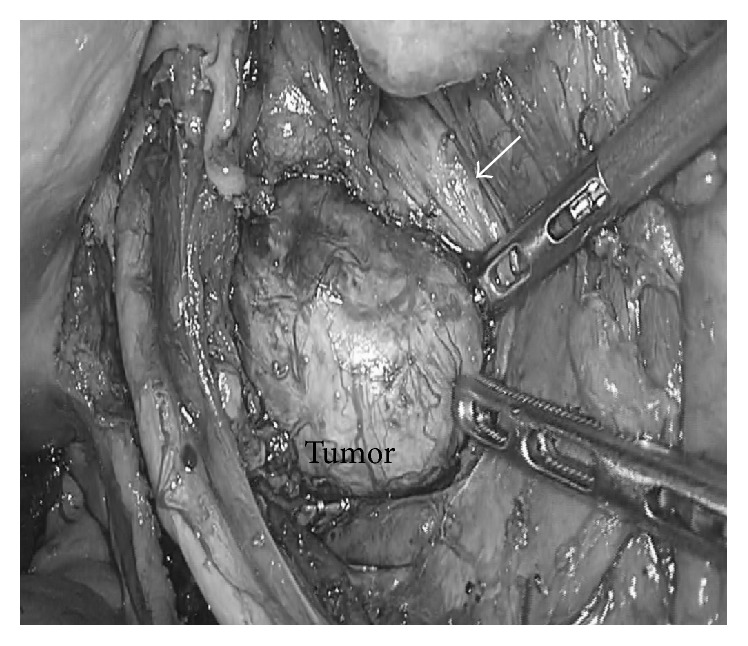
The tumor located at the dorsal side of the right sciatic nerve (arrow). The tumor originating from the right sciatic nerve.

**Figure 3:**
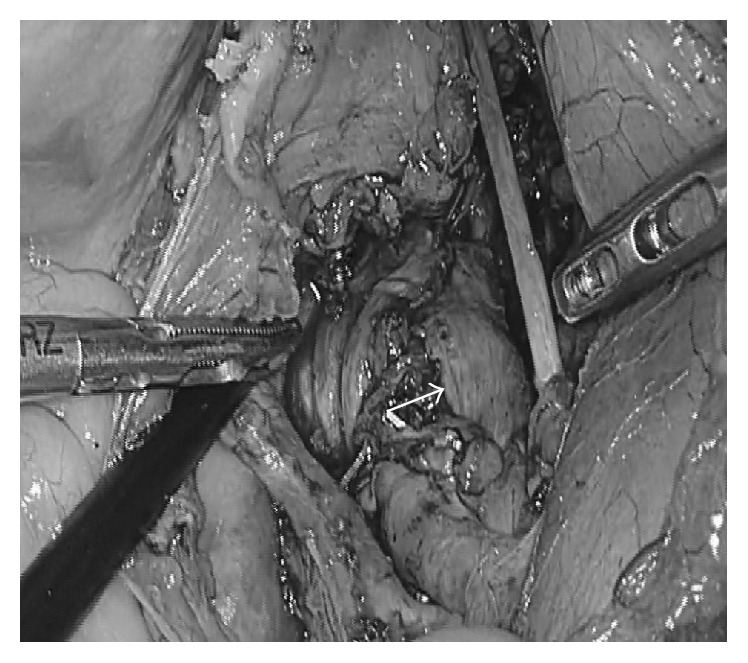
This was the surgical view with the preserved right sciatic nerve (arrow) after extirpation of the tumor.

We placed the patient in the lithotomy position under general anesthesia and inserted a ureter stent into the right ureter to prevent intraoperative injury. Next, we placed a 12 mm trocar with camera at the umbilicus using the open method. We then placed four 5 mm trocars at the bilateral upper and lower quadrants. The camera showed that the mass lesion (approximately 70 mm in diameter) covered the retroperitoneum in the right lateral pelvic space. We divided the right ureter and exposed the external iliac artery and vein. The tumor was located close to the right internal iliac artery and vein. We carefully isolated the tumor from the surrounding tissue using a THUNDERBEAT handheld system (Olympus Corporation, Japan). We dissected the obturator artery and vein to secure the surgical field. We resected the branches of the internal iliac vein as they were firmly adhered to the tumor. We carefully dissected the tumor from the surrounding tissues using both sharp and blunt maneuvers. The tumor was located at the dorsal side of the right sciatic nerve and was firmly adhered to the nerve (Figure 2). We suspected the mass to be a neurogenic tumor arising from the right sciatic nerve. The tumor was carefully isolated from the right sciatic nerve and freed from the surrounding tissues. We enlarged the umbilical incision to 4 cm and inserted a Smart Retractor (TOP Corporation, Japan). We removed the tumor through the enlarged incision covered by the Smart Retractor. No spillage occurred. After complete extirpation of the tumor, we preserved the right sciatic nerve in the right lateral pelvic space (Figure 3). Finally, we inserted a drain into the pouch of Douglas. The total operative time was 330 min, and total blood loss was 126 mL.

**Figure 4:**
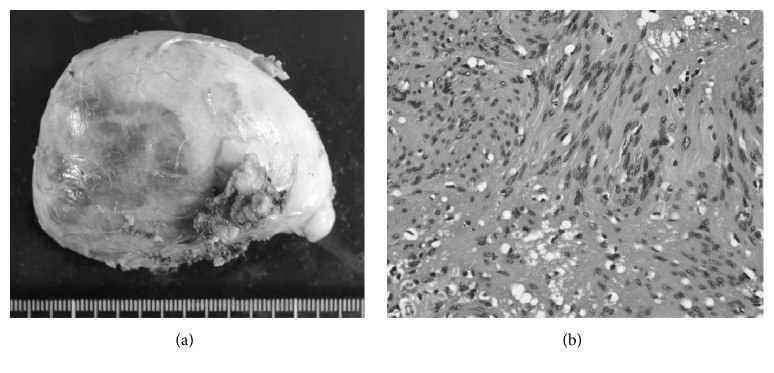
(a) The specimen was a firm, elastic, 70 × 50 mm mass with a capsule. (b) Palisade arrangement of spindle-shaped cells (hematoxylin-eosin, ×400).

On inspection, the specimen was a firm, elastic, 70 × 50 mm mass with a capsule (Figure 4(a)). In section, the mass was yellow and white in color, with a solid consistency. Pathological examination showed a fibrous capsule and a palisade arrangement of spindle-shaped cells originating from the Schwann cells (Figure 4(b)). We observed extensive degenerative change in the tumor. We made a pathological diagnosis of benign schwannoma.

The patient recovered well, but mild sciatic nerve palsy of right foot remained. She has continued rehabilitation training with a therapeutic orthosis.

## 3. Discussion

Pelvic schwannomas are rare, especially those originating from the sciatic nerve in the pelvic space. According to previous reports, schwannomas originating from the sciatic peripheral nerve in the foot can be resected percutaneously by an orthopedic surgeon [6]. Another report documented a giant abdominoperineal schwannoma that was treated surgically by a urologist [7]. As these reports show, surgeons from a range of disciplines can treat pelvic schwannomas. However, because the surgical approach to the lateral pelvic space was required in the present case, the operation was performed by a colorectal surgical team with the support of an orthopedic surgeon.

Preoperative diagnosis of schwannomas is difficult [1, 4]. US, CT, and MRI can visualize well-defined solid mass lesions, but these modalities are nonspecific in most cases. It has been reported that US or CT-guided fine needle aspiration biopsy is useful for preoperative diagnosis. However, malignancy cannot be excluded by the histological analysis of a specimen of tissue from a large tumor. Therefore, complete surgical resection for pelvic tumors might be the gold standard of treatment.

As the lateral pelvic space is narrow, approaching these tumors can be difficult during surgery. In open surgery, a large skin incision in the abdomen is required to resect a tumor in a lateral pelvic space. Recently, it has been reported that laparoscopic lateral lymph node dissection for locally advanced rectal cancers is safe and feasible [5]. In the present case, the laparoscopic approach provided a clear visual field with magnification, without the need for large skin incisions. This view was also very useful when dividing the schwannoma from the right sciatic nerve and dissecting the vessels adhered to the tumor. Robotic laparoscopic resection of a pelvic schwannoma has also been reported [8], and the delicate surgical technique afforded by this method may be very useful for the resection of neurogenic tumors while preserving the nerve.

Surgical resection of a schwannoma should aim to preserve the associated nerves. In the present case, although we preserved the nerve macroscopically, it was damaged microscopically. As previously reported, in cases where the schwannoma originated from a branch of the peripheral nerves, surgical damage to the nerve should not be symptomatic [9]. In the present case, however, the tumor originated from the main nerve trunk, and the insignificant damage to the nerve caused while attempting to preserve the main nerve trunk induced mild neurological disorder. The nature of the postoperative neurological deficit might depend on the primary site of the schwannoma [10]. In future, more delicate surgical techniques, such as robotic surgery, should be used to reduce neurological disorders following resection of schwannomas.
